# PLK-1 Regulation of Asymmetric Cell Division in the Early *C. elegans* Embryo

**DOI:** 10.3389/fcell.2020.632253

**Published:** 2021-01-21

**Authors:** Amelia J. Kim, Erik E. Griffin

**Affiliations:** Department of Biological Sciences, Dartmouth College, Hanover, NH, United States

**Keywords:** PLK1 (Polo-like Kinase 1), *C. elegans*, asymmetric cell division (ACD), cell polarity, PAR proteins

## Abstract

PLK1 is a conserved mitotic kinase that is essential for the entry into and progression through mitosis. In addition to its canonical mitotic functions, recent studies have characterized a critical role for PLK-1 in regulating the polarization and asymmetric division of the one-cell *C. elegans* embryo. Prior to cell division, PLK-1 regulates both the polarization of the PAR proteins at the cell cortex and the segregation of cell fate determinants in the cytoplasm. Following cell division, PLK-1 is preferentially inherited to one daughter cell where it acts to regulate the timing of centrosome separation and cell division. PLK1 also regulates cell polarity in asymmetrically dividing *Drosophila* neuroblasts and during mammalian planar cell polarity, suggesting it may act broadly to connect cell polarity and cell cycle mechanisms.

## Introduction

Asymmetric cell division is a process in which a dividing cell gives rise to daughter cells with differing fate, size and/or function. In bacteria and yeast, asymmetric divisions are widespread and give rise to cells that differ in morphology, function or replicative capacity (Macara and Mili, 2008; Kysela et al., 2013). In metazoans, asymmetric divisions contribute to the diversification of cell types during embryonic development and are also required to maintain tissue homeostasis in adults, for example through the continued asymmetric division of stem cells (Knoblich, 2010). While diverse molecular mechanisms control asymmetric divisions in unicellular and multicellular organisms (Macara and Mili, 2008), coordination between cell polarization and cell cycle progression lies at the heart of each asymmetric division: factors are polarized prior to cell division such that they are partitioned unequally to the daughter cells upon cytokinesis (Li, 2013; Venkei and Yamashita, 2018). Therefore, understanding the interplay between cell polarization, cell cycle and cell division mechanisms is central to understanding how cells divide asymmetrically (Knoblich, 2010; Noatynska et al., 2010).

Polo-like kinase 1 (PLK1) is a conserved and essential mitotic kinase that regulates centrosome duplication and maturation, mitotic entry, bipolar spindle formation, chromosome segregation, and cytokinesis (Barr et al., 2004; Petronczki et al., 2008; Archambault and Glover, 2009; Pintard and Archambault, 2018). PLK1 activity is relatively low during interphase and increases at the G2/M transition before falling again after anaphase. Abnormally high PLK1 activity during G2 and M-phase is associated with tumorigenesis, and has therefore been extensively studied as a target for cancer therapeutics (Strebhardt and Ullrich, 2006). In addition to its canonical mitotic functions, important roles for PLK1 in asymmetric cell division have been characterized. In this review, we focus on the role of PLK-1 during the asymmetric division of the one-cell *C. elegans* embryo (zygote). We describe the mechanisms by which PLK-1 both regulates the polarization of the worm zygote and as well as the ways in which the asymmetric inheritance of PLK-1 contributes to differences between daughter cells at the two-cell stage.

## Overview of PLK1 Structure and Regulation

**Figure 1:**
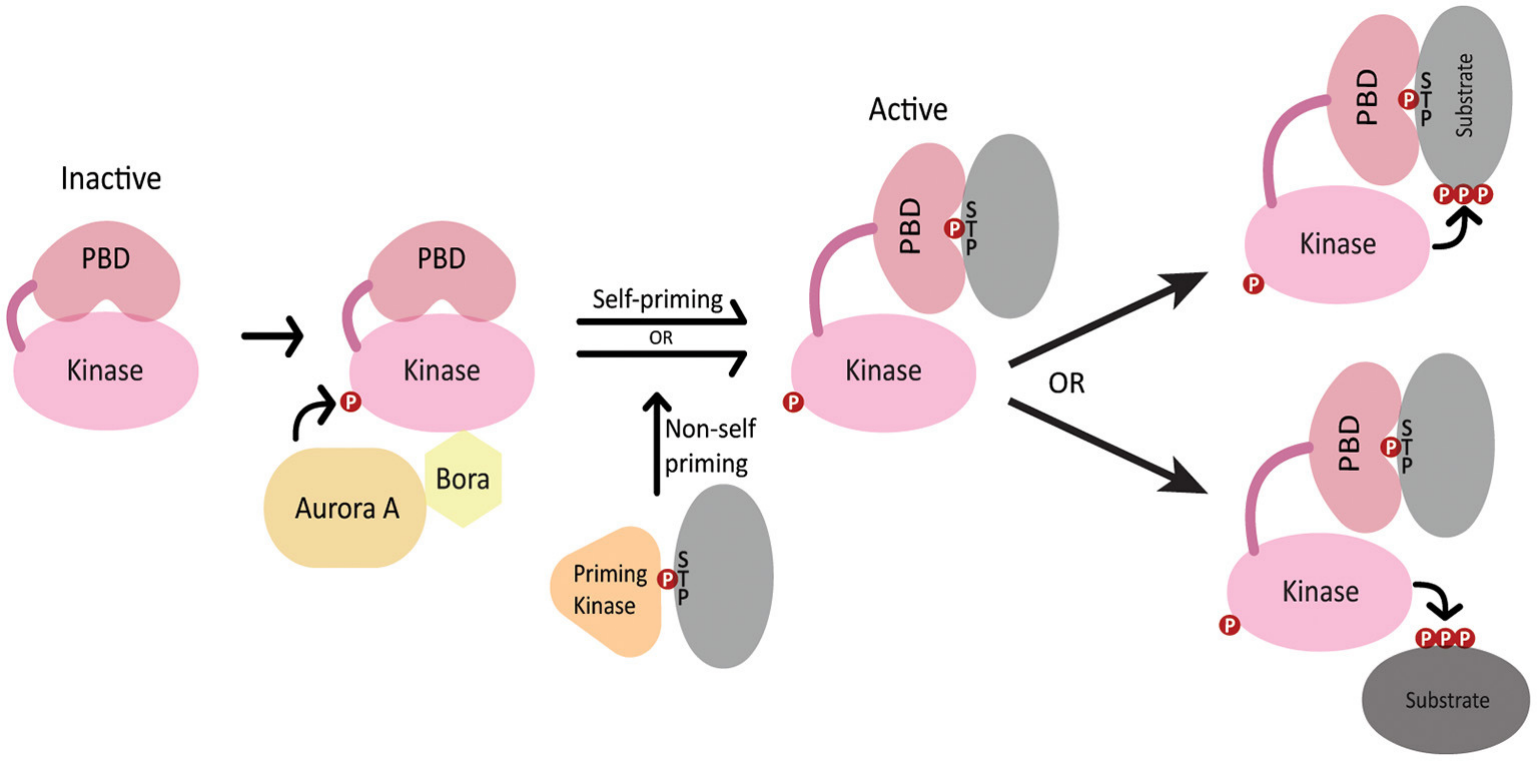
Regulation of PLK1 activation and localization. In the inactive state, the kinase domain activation loop is not phosphorylated and the PBD domain autoinhibits the kinase domain. Aurora A and SPAT/Bora stimulate kinase activity through phosphorylation of the PLK1 activation loop. The PBD domain binds phosphopeptides generated by PLK1 (self-primed) or by a priming kinase (non-self-primed), which relieves autoinhibition of the kinase domain. The PBD interacts either with PLK1 substrates or localizes PLK-1 in proximity to its substrates.

*polo* was first identified in *Drosophila* (Sunkel and Glover, 1988) and its homologs include *S. cerevisiae* Cdc5, *S. pombe* Plo1, *C. elegans* PLK-1 and human PLK1 (Archambault and Glover, 2009). PLK1 contains an N-terminal Serine/Threonine kinase domain that is highly conserved among polo-like kinases and is similar to those in Aurora kinases and calcium/calmodulin-dependent kinases (Zitouni et al., 2014). PLK1 kinase activity is stimulated by phosphorylation of the activation loop in the PLK1 kinase domain by Aurora A kinase, which depends on the Aurora A cofactor Bora (Jang et al., 2002; Macurek et al., 2008; Seki et al., 2008; Tavernier et al., 2015b) (Figure 1). The C-terminus features a non-catalytic Polo box domain (PBD) that binds phosphopeptides generated by either PLK1 itself (self-primed) or by other kinases (non-self primed) (Zitouni et al., 2014) (Figure 1). The interaction between the PBD and various binding partners guides PLK1 localization to different cellular structures such as the centrosome, kinetochores, nuclear envelope and midbody (Lee et al., 1998; Elia et al., 2003). Additionally, the interaction between the PBD and its binding partners relieves the autoinhibition of PLK1 kinase activity by the intramolecular interaction between the PBD and the kinase domain, thereby coupling PLK1 localization with its activation toward specific substrates (Elia et al., 2003; Lowery et al., 2005; Zitouni et al., 2014) (Figure 1).

## Introduction to the Asymmetric Division of the *C. elegans* Embryo

The one-cell *C. elegans* embryo undergoes an asymmetric division to give rise to an anterior daughter cell named AB and a posterior daughter cell named P1. AB and P1 differ in several respects: AB gives rise exclusively to somatic lineages whereas P1 gives rise to both somatic and germline lineages, AB is larger than P1, and AB divides roughly 2 min before P1 with a mitotic spindle oriented orthogonally to the P1 spindle (Rose and Gonczy, 2014; Griffin, 2015). All aspects of this asymmetric division depend on the polarization of the embryo by the conserved PAR polarity regulators.

The PAR proteins include the Anterior PAR proteins (aPARs) PAR-3, PAR-6 and PKC-3/aPKC and the Posterior PAR proteins (pPARs) PAR-1 and PAR-2. The aPARs and pPARs antagonize each other's cortical association such that any region of the cortex is typically occupied by either the aPARs or the pPARs, but not both (Lang and Munro, 2017). The PAR proteins are deposited in the oocyte and remain symmetrically distributed as the oocyte matures and as the newly fertilized embryo completes meiosis II. Upon the completion of meiosis, several symmetry breaking cues at the posterior pole combine to trigger the precisely timed, robust, and rapid polarization of the embryo (Goldstein and Hird, 1996). These cues include microtubules and Aurora A kinase activity that emanates from the sperm-donated centrosome at the posterior end (Tsai and Ahringer, 2007; Motegi et al., 2011; Klinkert et al., 2019; Zhao et al., 2019) and redox signaling from mitochondria near the posterior cortex (De Henau et al., 2020). These cues trigger the establishment of polarity, which takes roughly 10 min and results in the concentration of the aPARs at the anterior cortex and the pPARs at the posterior cortex. During polarity establishment, anteriorly-directed cortical actomyosin flows help to sweep the cortical aPARs out of the posterior domain, thereby allowing pPARs to load from the cytoplasm onto the posterior cortex (Cheeks et al., 2004; Munro et al., 2004; Goehring et al., 2011; Gubieda et al., 2020). These asymmetries are maintained for ~10 min until the embryo divides. From their polarized domains, the PAR proteins regulate the polarization of both cortical and cytoplasmic factors along the anterior/posterior (A/P) axis that result in the differences in size, fate, spindle orientation and cell division timing between AB and P1.

**Figure 2:**
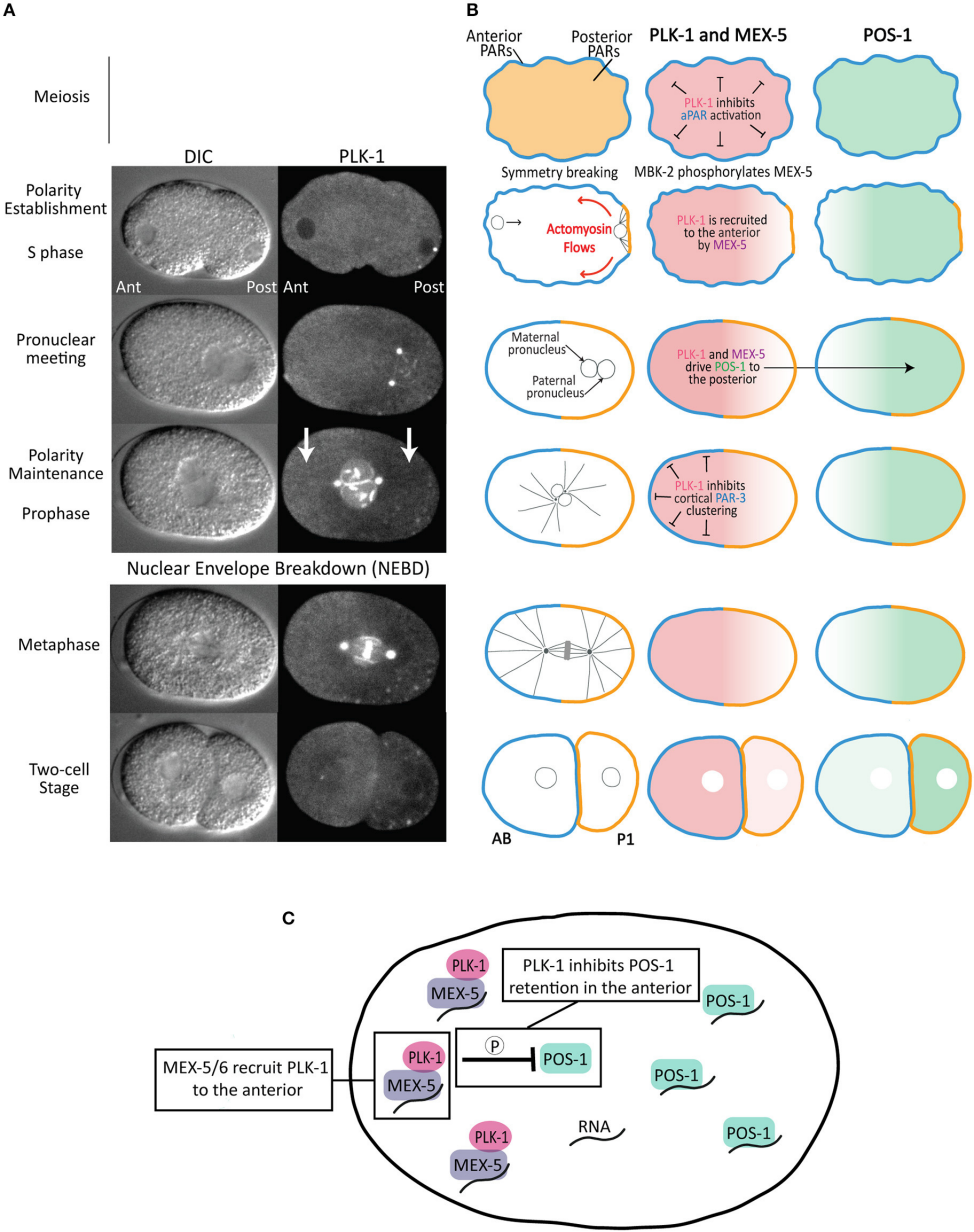
Asymmetric division of the *C. elegans* zygote. **(A)** DIC and fluorescence images of a one-cell embryo expressing PLK-1::sGFP (Martino et al., 2017) from polarity establishment through its asymmetric division. Note that, in addition to its localization to the nuclear envelope, centrosomes and chromosomes, there is a cytoplasmic pool that is enriched in the anterior cytoplasm relative to the posterior cytoplasm (indicated by white arrows). **(B)** Schematics of aPAR (blue), pPAR (yellow), PLK-1, MEX-5 and POS-1 localization during the asymmetric division of the zygote. The stages are as indicated in **(A)**. The left panels indicate the position of the maternal and paternal pro-nuclei (black circles), microtubules (black lines) and chromosomes (gray) in the mitotic spindle. **(C)** Model for the mechanism by which MEX-5 and PLK-1 regulate POS-1 segregation. MEX-5 and PLK-1 are retained in the anterior cytoplasm, likely through the association of MEX-5 with RNA. PLK-1 phosphorylates POS-1 to inhibit its retention in the anterior. As a consequence, POS-1 enriches in the posterior cytoplasm, presumably on RNA. This model predicts POS-1 dephosphorylation is required for its retention in the posterior.

Consistent with its canonical role in the cell cycle, PLK-1 regulates meiotic and mitotic progression in the *C. elegans* zygote (Chase et al., 2000), including by promoting nuclear envelope breakdown (Rahman et al., 2015, 2020; Martino et al., 2017; Velez-Aguilera et al., 2020) and centrosome maturation (Woodruff et al., 2015). Here, we focus on PLK-1's contributions to the asymmetric division of the zygote as both a regulator at multiple stages of the zygote polarization and as a factor whose asymmetric inheritance contributes directly to differences between AB and P1 (Figures 2A,B). It is important to keep in mind that because complete depletion of PLK-1 activity results in sterility, the early embryonic functions of PLK-1 described below reflect the phenotypes of embryos partially depleted of PLK-1 activity.

## PLK-1 Regulates Polarization of the *C. elegans* Zygote

### PLK-1 Suppresses Premature Polarization During Meiosis

The *C. elegans* oocyte remains apolar as it matures, is fertilized and completes two meiotic divisions. In the maturing oocyte, the pPARs occupy the cell cortex and the aPARs are restricted to the cytoplasm. By anaphase II of meiosis, the PAR proteins have switched locations with the aPARs at the cell cortex and the pPARs restricted to the cytoplasm (Reich et al., 2019), positioning them to respond to the posterior symmetry breaking cues upon the completion of meiosis (Figure 2B). PLK-1 and Aurora A (AIR-1) suppress the premature cortical loading of the anterior PARs, thereby ensuring polarization is only established following the completion of meiosis. Depletion of either AIR-1 or PLK-1 results in the premature cortical localization of aPARs and the restriction of the pPARs to the cytoplasm in oocytes and meiotic embryos. This recruitment of the aPARs to the cortex “activates” the PAR polarity system prematurely (Figure 2B), such that normally cryptic symmetry breaking cues present in the meiotic embryo, including regions of high membrane curvature and microtubules associated with the meiotic spindle, can trigger polarization before the completion of meiosis (Wallenfang and Seydoux, 2000; Kapoor and Kotak, 2019; Klinkert et al., 2019; Reich et al., 2019; Zhao et al., 2019). As a result, *air-1* and *plk-1* depleted embryos display a range of polarity defects, including bipolarity, reversed polarity and mispositioned PAR domains (Noatynska et al., 2010; Klinkert et al., 2019; Reich et al., 2019; Zhao et al., 2019). Suppressing activation of the PAR system until after the completion of meiosis ensures the embryo only polarizes in response to the multiple coordinate symmetry breaking cues from the posterior end, resulting in the rapid and highly stereotyped establishment of the anterior/posterior polarity axis.

In the future, it will be important to determine whether PLK-1 and/or AIR-1 is the principal inhibitor of PAR network activation. Depletion of SPAT/Bora, which facilitates the activation of PLK-1 by AIR-1, results in similar polarity defects as depletion of either AIR-1 or PLK-1 (Noatynska et al., 2010). This observation is consistent with PLK-1 acting downstream of AIR-1 to suppress activation of the PAR network. Additionally, it will be important to identify the relevant substrates and mechanisms that suppress activation. As discussed in the next section, PLK-1 phosphorylates PAR-3 to suppress its cortical localization in mitotic one-cell embryos (Dickinson et al., 2017), suggesting this could be one possible mechanism by which PLK-1 might suppress aPAR cortical loading in oocytes and meiotic embryos.

### PLK-1 Disassembles PAR-3 Clusters During Polarity Maintenance

Polarity establishment coincides with the dramatic flow of the contractile actomyosin cortex from the posterior toward the anterior (Figure 2B). These actomyosin flows help move the aPARs out of the posterior, clearing the way for pPARs to load from the cytoplasm onto the posterior cortex (Munro et al., 2004; Goehring et al., 2011). During actomyosin flows, the aPARs colocalize in prominent cortical clusters that stabilize their cortical association and help entrain them within actomyosin flows, thereby promoting segregation to the anterior (Tabuse et al., 1998; Hung and Kemphues, 1999; Beers and Kemphues, 2006; Sailer et al., 2015; Dickinson et al., 2017; Rodriguez et al., 2017; Wang et al., 2017). PAR-3 is critical for aPAR clustering: PAR-3 forms clusters in the absence of either PKC-3 or PAR-6, is required for PKC-3 clustering and contains an oligomerization domain that mediates its clustering (Dickinson et al., 2017; Rodriguez et al., 2017; Wang et al., 2017). Actomyosin flows cease when they reach roughly the midpoint of the A/P axis and shortly thereafter PAR-3 clusters disperse as the embryo enters mitosis and the maintenance phase of polarization.

The assembly of PAR-3 into cortical clusters is inhibited by PLK-1. In *plk-1(RNAi)* embryos, PAR-3 clusters form prematurely (before symmetry breaking), potentially reflecting the premature activation of the PAR network in meiotic embryos discussed above (Dickinson et al., 2017; Reich et al., 2019) (Figure 2B). Additionally, PAR-3 clusters fail to disassemble during maintenance phase in *plk-1* mutant embryos. The PAR-3 N-terminus contains two putative PBD binding motifs, both of which are required for the PLK-1 PBD domain to bind PAR-3 in a yeast two-hybrid assay. This interaction also depends on the phosphopeptide-binding motif in the PLK-1 PBD domain, suggesting that priming phosphorylation of PAR-3 stimulates an interaction between PLK-1 and PAR-3 (Dickinson et al., 2017). PAR-3 contains two putative PLK-1 phosphorylation sites (Thr32 and Thr89), one of which was shown to be phosphorylated by PLK-1 *in vitro*. Importantly, phosphomimetic substitutions at these residues prevent both PAR-3 oligomerization and PAR-3 localization to the cell cortex (Dickinson et al., 2017). Taken together, these data suggest that relatively low levels of PLK-1 activity during interphase allows PAR-3 cluster formation and entrainment in actomyosin flows during polarity establishment. PLK-1 activity increases at the transition from establishment to maintenance phase, at which point PLK-1 phosphorylates PAR-3 to disperse aPAR clusters (Figure 2B). This dispersal makes PAR-3 insensitive to subsequent actomyosin flows during cytokinesis (Dickinson et al., 2017). In the future, it will be interesting to learn how PLK-1 activity toward PAR-3 is coordinated with the transition from polarity establishment to polarity maintenance. For example, the global increase in PLK-1 activity as the embryo enters mitosis could trigger PAR-3 cluster disassembly. Alternately, there may be unknown mechanisms that temporally control PLK-1 activity specifically toward PAR-3. Consistent with the latter possibility, PLK-1 acts prior to maintenance phase to drive the segregation of the cytoplasmic fate determinant POS-1, as discussed below (Han et al., 2018).

### PLK-1 Controls Segregation of POS-1

A key output of the PAR polarity system is to control the segregation of cytoplasmic cell fate determinants along the A/P axis of the one-cell embryo, leading to the preferential partitioning of somatic factors to AB and germline factors to P1 (Figure 2B). The polarization of cytoplasmic proteins is not caused by local protein degradation or local protein synthesis, but rather by their preferential retention in either the anterior or posterior cytoplasm, which leads to their accumulation in that cytoplasmic domain (Tenlen et al., 2008; Daniels et al., 2009, 2010; Griffin et al., 2011; Wu et al., 2015, 2018). The posterior kinase PAR-1 inhibits the retention of its substrate the RNA-binding protein MEX-5 in the posterior cytoplasm, leading to MEX-5 segregation to the anterior cytoplasm (Pagano et al., 2007; Tenlen et al., 2008; Griffin et al., 2011). In turn, MEX-5, along with the highly similar protein MEX-6 (MEX-5/6 hereafter), controls the segregation of germline factors to the posterior cytoplasm by inhibiting their retention in the anterior (Schubert et al., 2000) (Figure 2B). For example, MEX-5/6 inhibit the retention of the RNA-binding protein POS-1 in the anterior, leading to the progressive accumulation of POS-1 in the posterior (Farley et al., 2008; Wu et al., 2015; Han et al., 2018). Both the retention of MEX-5 (and presumably MEX-6) in the anterior and the retention of POS-1 in the posterior depends on their ability to bind RNA (Griffin et al., 2011; Han et al., 2018), suggesting they may accumulate on RNA in the anterior and posterior cytoplasm, respectively (Figure 2C).

The ability of MEX-5/6 to drive POS-1 segregation to the posterior depends on PLK-1. The priming kinase MBK-2 phosphorylates MEX-5 and MEX-6 (on MEX-5 residue Thr186), generating a binding site for the PBD domain of PLK-1 (Nishi et al., 2008). MBK-2 becomes active upon the completion of meiosis, suggesting the interaction between MEX-5 and PLK-1 is likely coupled to this transition and to the onset of polarization (Stitzel et al., 2006, 2007; Maruyama et al., 2007; Cheng et al., 2009). As a consequence of its interaction with MEX-5/6, the cytoplasmic pool of PLK-1 becomes enriched in the anterior cytoplasm (Nishi et al., 2008) (Figures 2A,B). In addition, binding to MEX-5 increases PLK-1 kinase activity *in vitro*, likely by relieving PLK-1 autoinhibition by the PBD domain (Nishi et al., 2008). Similar to *mex-5/6* mutant embryos, POS-1 segregation fails in embryos in which PLK-1 has been depleted, in which PLK-1 kinase activity has been inhibited or in embryos in which the interaction between PLK-1 and MEX-5 has been disrupted through mutation of the priming phosphorylation site on MEX-5 (Thr186) (Han et al., 2018). *In vitro*, PLK-1 phosphorylates a cluster of residues in the POS-1 C-terminal region. *In vivo*, a non-phosphorylatable allele of POS-1 fails to segregate because it is inappropriately retained in the anterior cytoplasm. In contrast, a phosphomimetic allele of POS-1 fails to segregate because it is not retained in the posterior cytoplasm (Han et al., 2018). These data suggest MEX-5/PLK-1 complexes in the anterior cytoplasm phosphorylate POS-1 to inhibit its retention in the anterior (Figure 2C). Because RNA-binding is required for MEX-5 retention in the anterior and for POS-1 retention in the posterior, one possibility is that MEX-5 recruits PLK-1 to RNA in the anterior where it is positioned to inhibit POS-1 retention on RNA. In contrast to PLK-1 regulation of PAR-3 disassembly, PLK-1 regulates POS-1 segregation throughout both polarity establishment and maintenance phases, suggesting that at least the cytoplasmic pool of PLK-1 is active during interphase.

POS-1 is one of a collection of germline RNA-binding proteins that segregate to the posterior cytoplasm in response to MEX-5/6 (Schubert et al., 2000). Many of these germline proteins concentrate in germ granules (called P granules in *C. elegans*) which are non-membranous, phase-separated condensates composed primarily of RNA and RNA-binding proteins (Updike and Strome, 2010; Seydoux, 2018). The relatively high concentration of MEX-5/6 in the anterior cytoplasm causes P granules to disassemble, whereas P granules assemble and grow in the posterior cytoplasm (Brangwynne et al., 2009). One mechanism that disassembles P granules in the anterior is the sequestration of RNA by MEX-5/6, which starves P granule proteins of the RNA they need to assemble in granules (Saha et al., 2016; Smith et al., 2016). Interestingly, there is genetic evidence that PLK-1 may also act with MEX-5/6 to stimulate P granule disassembly: both depletion of PLK-1 by RNAi and mutation of the PLK-1 binding site on MEX-5 results in the inappropriate stabilization of P granules in the anterior cytoplasm (Nishi et al., 2008; Wu et al., 2019). Whether PLK-1 plays a direct role in P granule disassembly, for example by inhibiting P granule assembly through direct phosphorylation of P granule protein(s), awaits further study.

## PLK-1 Asymmetry Contributes to Differences Between AB and P1

In addition to the role of PLK-1 in regulating asymmetries before the division of the one cell embryo, the asymmetric inheritance of PLK-1 following cell division also contributes to differences in the timing of centrosome separation and cell division between AB and P1. The preferential inheritance of PLK-1 to AB derives from the interaction between PLK-1 and MEX-5 and the resultant enrichment of PLK-1 in the anterior cytoplasm before cell division (Nishi et al., 2008).

### PLK-1 Regulation of Cell Division Asymmetry

**Figure 3:**
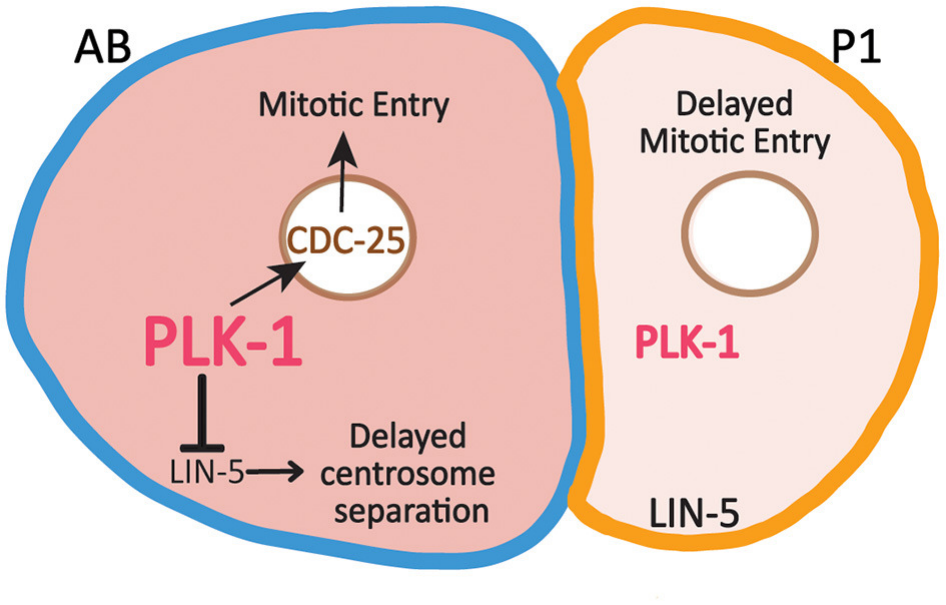
Asymmetric inheritance of PLK-1 contributes to differences in the timing of cell division and centrosome separation between AB and P1. Relatively high levels of PLK-1 in AB promotes mitotic entry by increasing nuclear levels of CDC25, which contributes to the faster cell division timing in AB relative to P1. PLK-1 also acts to reduce cortical LIN-5 levels in AB, which delays centrosome separation in AB.

As in many animal embryos, *C. elegans* development begins with a series of rapid, reductive cleavages that alternate between interphase and mitosis and lack G1 and G2 phases. Beginning at the two-cell stage, cells in the germline P lineage divide more slowly than their somatic sister cells. For example, P1 divides roughly 2 min after AB (Noatynska et al., 2013; Tavernier et al., 2015a) as a result of the combined effect of differences in the activities of several cell cycle regulators. Part of the cell division asynchrony is due to delayed DNA replication in P1 and the preferential activation of the ATL-1/CHK-1 DNA replication checkpoint in P1, which delays P1's entry into mitosis (Brauchle et al., 2003; Benkemoun et al., 2014). In addition, the preferential inheritance of both PLK-1 and cyclin B3 promotes the advanced timing of AB division (Budirahardja and Gönczy, 2008; Rivers et al., 2008; Michael, 2016). PLK-1 promotes mitotic entry in part through phosphorylation and activation of CDC25. Indeed, the enrichment of PLK-1 in AB correlates with higher levels of nuclear CDC25 in AB than in P1 (Rivers et al., 2008) (Figure 3). Partial depletion of either PLK-1 or CDC25 causes a more substantial delay in the division of P1 than the division of AB, consistent with the idea that the low levels of PLK-1 and CDC25 in P1 are limiting (Budirahardja and Gönczy, 2008; Rivers et al., 2008).

### PLK-1 Regulation of Centrosome Separation in AB

In addition to promoting mitotic entry, the enrichment of PLK-1 in AB also delays centrosome separation in AB relative to P1 (Figure 3). LIN-5 (the *C. elegans* NUMA ortholog) is a dynein binding protein present in the cytoplasm and at the cell cortex. PLK-1 acts to reduce the cortical levels of LIN-5 in AB relative to P1 (Bondaz et al., 2019). LIN-5 promotes the separation of centrosomes by exerting force on centrosomes through astral microtubules that reach the cortex. As a result of the differences in cortical LIN-5 levels, centrosome separation is delayed in AB relative to P1. Embryos depleted of KLP-7 (the *C. elegans* MCAK ortholog) are particularly sensitive to the low levels of cortical LIN-5 in AB and therefore exhibit enhanced delay in the timing of centrosome in AB. This delay can be suppressed through depletion in PLK-1 which increases cortical LIN-5 levels in AB (Bondaz et al., 2019). In mammalian cells, PLK1 regulates cortical NUMA levels directly, suggesting PLK-1 may directly target LIN-5 in AB to reduce its cortical association (Kiyomitsu and Cheeseman, 2012; Sana et al., 2018).

## Conclusion

In addition to its role in the asymmetric division of the *C. elegans* zygote, PLK1 also contributes to asymmetric division and cell polarization in other cell types. During the asymmetric division of *Drosophila* neuroblasts, the asymmetric localization of cortical cell fate determinants is regulated by Polo (the *C. elegans* ortholog of PLK-1). The membrane-associated proteins Pros, Numb, Miranda and Partner of Numb (Pon) are uniformly distributed during interphase. During late prophase, Polo directly phosphorylates Pon, leading to the asymmetric localization of PON and its binding partner Numb to the basal cortex (Wang et al., 2007). In *polo* mutants, Pon and Numb are not asymmetrically localized to basal cortex, causing a failure of asymmetric division that leads to neuroblast over-proliferation. Additionally, increased Polo activity at the daughter centrosomes relative to mother centrosomes is essential for the retention of the daughter centrosome in the neuroblast cell following cell division (Januschke et al., 2013; Conduit et al., 2014; Gallaud et al., 2020). The extent to which the resulting asymmetric inheritance of mother and daughter centrosomes regulates the fate of apical and basal daughter cells remains an open area of investigation. Similarly, during the asymmetric division of *S. cerevisiae* cells, increased Cdc5 activity at the mother centrosome (spindle pole body) is essential for its asymmetric inheritance to the newly born daughter cell (Maekawa et al., 2017). Non-random inheritance of mother and daughter centrosomes is widespread and may contribute to the partitioning of aging/rejuvenation programs between daughter cells or in the asymmetric inheritance of centrosome-associated fate determinants (Macara and Mili, 2008; Pelletier and Yamashita, 2012; Manzano-Lopez et al., 2019; Sunchu and Cabernard, 2020). PLK1 has also been shown to regulate planar cell polarity in mammalian epithelial cells through phosphorylation of CELSR-1 (Flamingo in *Drosophila*), which triggers CELSR-1 clearance from the cell surface as cells prepare to undergo cytokinesis (Shrestha et al., 2015). These studies, along with the numerous ways in which PLK-1 regulates the asymmetric division of the *C. elegans* zygote, raise the possibility that PLK1 may act broadly and in diverse ways to control both cell polarization and cell cycle progression. In the future, it will be interesting to learn in which contexts PLK1's role in cell polarization is coordinated with its role in cell cycle progression and in which contexts PLK1 acts independently in these two processes.

## Author Contributions

All authors listed have made a substantial, direct and intellectual contribution to the work, and approved it for publication.

## Conflict of Interest

The authors declare that the research was conducted in the absence of any commercial or financial relationships that could be construed as a potential conflict of interest.
